# Long-Term Incidence of Advanced Colorectal Neoplasia in Patients with Serrated Polyposis Syndrome: Experience in a Single Academic Centre

**DOI:** 10.3390/cancers13051066

**Published:** 2021-03-03

**Authors:** Daniel Rodríguez-Alcalde, Guillermo Castillo-López, Jorge López-Vicente, Luis Hernández, Mercedes Lumbreras-Cabrera, Diego Moreno-Sánchez

**Affiliations:** 1 Digestive Disease Section, Hospital Universitario de Móstoles, 28935 Móstoles, Spain; guillermo.castillo@salud.madrid.org (G.C.-L.); jlvicente@salud.madrid.org (J.L.-V.); lhernandezvi@saludcastillayleon.es (L.H.); mercedes.lumbreras@salud.madrid.org (M.L.-C.); dmorenos@salud.madrid.org (D.M.-S.); 2 Digestive Disease Section, Hospital Santos Reyes, 09400 Aranda de Duero, Spain

**Keywords:** serrated polyposis syndrome, colorectal cancer, advanced colorectal neoplasia, serrated polyp

## Abstract

**Simple Summary:**

Serrated polyposis syndrome is characterized by the development of large and/or multiple serrated polyps throughout the colorectum and is associated with an increased risk of colorectal cancer (CRC). Even though CRC incidence is low under adequate endoscopic follow-up, a substantial risk of advanced neoplasia (AN) has been described. Nevertheless, very few studies have focused on long-term surveillance. The main aim of this study was to evaluate the incidence of AN in a single-centre cohort followed over 10 years. Within endoscopic surveillance we did not find any CRC and we observed that five-year cumulative incidences of AN were much lower than in other studies. However, a significant reduction of these incidences during follow-up was not proven. Individuals at higher risk of AN were those who fulfilled both 2010 WHO criteria I and III. Our results suggest that at least patients at lower risk might benefit from the extension of surveillance intervals.

**Abstract:**

Serrated polyposis syndrome (SPS) implies a slightly elevated risk of colorectal cancer (CRC) during endoscopic follow-up, but its natural course is still not well known. The main objective of this study was to describe the long-term risk of developing advanced neoplasia (AN) in these patients. Until October 2020, individuals who fulfilled 2010 WHO criteria I and/or III for SPS were retrospectively recruited. We selected those under endoscopic surveillance after resection of all lesions >3 mm in a high-quality colonoscopy. We excluded patients with total colectomy at diagnosis and those with any interval between colonoscopies >3.5 years. We defined AN as advanced serrated polyp (≥10 mm and/or with dysplasia), advanced adenoma, or CRC. In 109 patients, 342 colonoscopies were performed (median = 3, median interval = 1.8 years) during a median follow-up after colonic clearance of 5.0 years. Five-year cumulative incidences of AN were 21.6% globally, and 5.6%, 10.8%, and 50.8% in patients who fulfilled criterion I, III, and both, respectively (*p* < 0.001). No CRC was diagnosed and only 1 (0.9%) patient underwent surgery. In conclusion, cumulative incidences of AN could be lower than previously described, at least in patients who fulfil the 2010 WHO criterion III alone. Therefore, low-risk individuals might benefit from less stringent surveillance.

## 1. Introduction

Serrated polyposis syndrome (SPS) is a condition characterized by the development of large and/or multiple serrated polyps (SPs) in the colon and rectum, which implies a higher risk of colorectal cancer (CRC) compared to the average-risk population [1,2,3,4,5,6]. In 2010 the World Health Organization (WHO) defined SPS based on the compliance of at least one of these criteria: (I) the presence of ≥5 SPs proximal to the sigmoid colon, with two or more being ≥10 mm in size; (II) the presence of any number of SPs occurring proximal to the sigmoid colon in a patient with a first-degree relative with SPS; and (III) the presence of more than 20 SPs spread throughout the colon [7]. These criteria were updated by the WHO in 2019: (I) the presence of ≥5 SPs proximal to the rectum, all ≥5 mm in size, with two or more being ≥10 mm in size; and (II) the presence of more than 20 SPs of any size spread throughout the colon, with ≥5 proximal to the rectum [8]. Previously considered rare [9,10,11], SPS has recently appeared as the most frequent colorectal polyposis syndrome. In the UK in a guaiac faecal occult blood test-based CRC screening program its prevalence was reported between 0.03% and 0.66%, whereas in colonoscopy-based programs it ranged from 0.1 to 0.4% [12,13]. Furthermore, Rivero-Sánchez et al. showed that, in a faecal immunochemical test (FIT)-based program, its prevalence rises up to 0.9% after one-year reassessment colonoscopy in patients with proximal SPs [14].

Initial small case series reported the lifetime CRC risk in individuals with SPS to be as high as 43–70% [9,10,15,16], which is very likely due to ascertainment bias. Therefore, in the absence of high-quality evidence, some authors recommended endoscopic surveillance every 1–3 years [17,18,19] and, in 2012, the US Multi-Society Task Force on Colorectal Cancer advised an annual colonoscopy for all patients with SPS [20]. However, two large cohort studies published in the last years have estimated much lower CRC risks in these patients, at levels of 15.8% and 29.3% respectively [21,22]. Beside this, once colonic clearance is achieved and under adequate endoscopic surveillance, CRC risk seems to be only slightly elevated. Two retrospective studies showed five-year cumulative incidences of 6.4% and 7% respectively [6,23] and no CRC was diagnosed in a prospective protocol with annual colonoscopy [24]. These studies have also shown that the risk of CRC and advanced neoplasia (AN) depends on endoscopic and pathological variables (proximal sessile serrated lesions, SPs with cytological dysplasia, advanced adenomas, advanced SPs at baseline, fulfilment of both WHO criteria I and III, etc.), and thus follow-up could be personalized according to individual risk. Recently, Bleijenberg et al. conducted a prospective surveillance protocol with an interval between colonoscopies of either one or two years depending on polyp burden, observing a low incidence of CRC and that the risk of AN was not increased in individuals recommended a two-year interval [25]. Based on all these findings, the European Society of Gastrointestinal Endoscopy (ESGE) now recommends endoscopic surveillance of SPS every one or two years [26], using the same criteria described by Bleijenberg et al. However, the natural course of this syndrome is still not well known because very few studies have focused on long-term follow-up. This makes necessary a more precise knowledge of the disease to better stratify patients according to their actual risk. This would help to minimize interval CRC and to reduce the colonoscopy burden that SPS represents nowadays, as a result of its relatively high prevalence and the intensive endoscopic follow-up advised.

Our group recently published a large multicentre study, with a median follow-up after clearing colonoscopy of 2.2 years, observing a three-year cumulative incidence of AN of 42.0% [23]. We analyse here the subgroup of patients followed in the Hospital Universitario de Móstoles, diagnosed according to 2010 WHO criteria for SPS and enrolled using the same principles as the aforementioned work [23]. This study aimed to describe the incidence of advanced colorectal neoplasia in patients with SPS within a much longer surveillance period, and their management in a single academic hospital.

## 2. Results

We recruited a total of 129 patients who fulfilled the 2010 WHO I and/or III criteria for SPS. Sixty (46.5%) of them were female and the mean age at SPS diagnosis was 59.9 years (SD = 9.1). One hundred and twelve (86.8%) had a smoking history—54 active and 58 prior to recruitment—and the mean body mass index was 27.9 (SD = 5.0), with 93 (72.1%) overweight/obese patients.

In the whole cohort 11 colorectal cancers (8.5%) were diagnosed, all of them before starting endoscopic surveillance, and only two since 2014. They were more frequent in men (*n* = 9; 81.8%) and detected at a mean age of 59.8 years (SD = 5.8). Regarding SPS phenotype, 1 (9.1%), 4 (36.4%), and 6 (54.5%) of these patients fulfilled criterion I, III, and both, respectively. Two out of 129 (1.6%) patients underwent total colectomy at SPS diagnosis, one due to CRC and the other because of severe polyposis.

**Figure 1:**
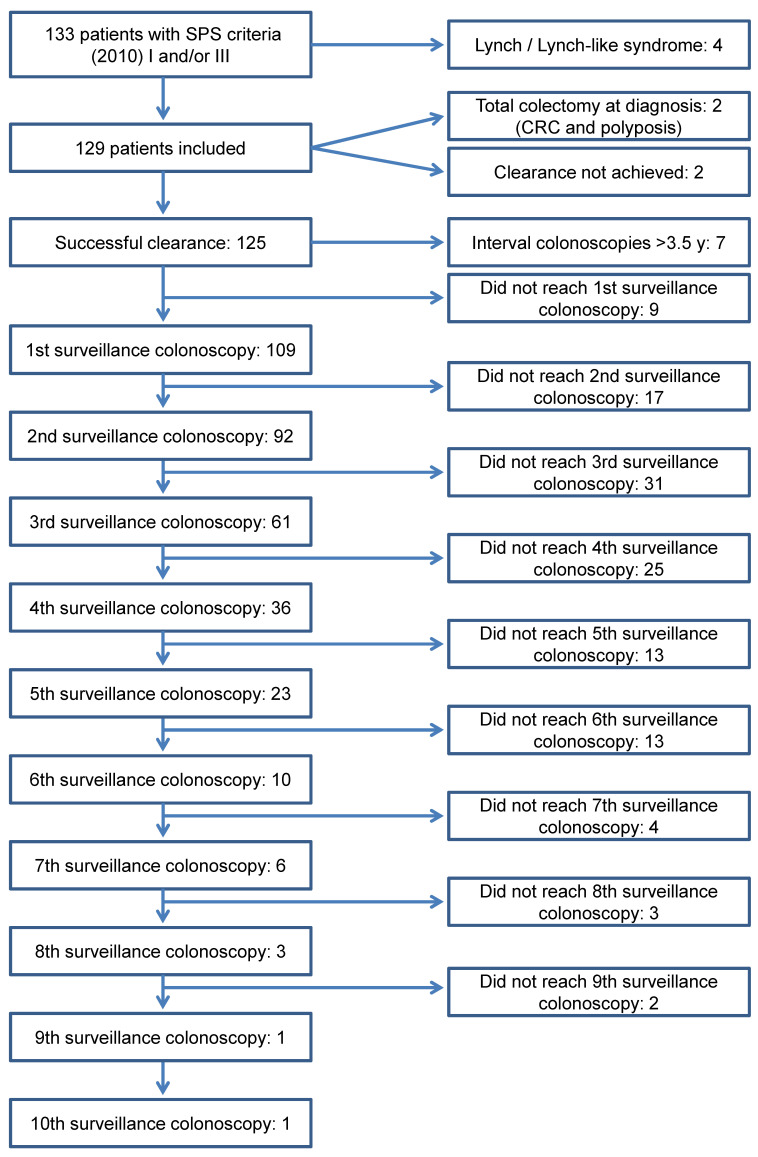
Flow chart of patients during the study period.

One hundred and nine individuals started endoscopic surveillance every 1–3 years after a successful colonic clearance and were included in the primary analysis of the study (Figure 1). Baseline characteristics of the population under follow-up are detailed in Table 1. Nine (8.3%) of these patients had a partial colonic resection before entering surveillance, eight because of CRC and one due to unresectable polyps; the remaining CRC was treated endoscopically, since it was a T1 in a pedunculated adenoma fulfilling all favourable histological criteria. There was no statistical association between age at SPS diagnosis and colonic phenotype. As regards gender, men fulfilled more frequently criterion III (75.0 vs. 35.8%) whereas criteria I and I + III predominated among women (24.5 vs. 8.9% and 39.6 vs. 16.1%, respectively; *p* < 0.001).

We performed a total of 342 surveillance colonoscopies with a median follow-up after clearance of 5.0 years (IQR = 3.3–7.2), resecting 2097 polyps—most of them hyperplastic (Table 2).

Eighteen (16.5%), 6 (5.5%), and 22 (20.2%) patients presented one or more advanced SPs, advanced adenoma, and AN during endoscopic surveillance, respectively, and no incident CRC was diagnosed. The per-patient incidence of lesions at each year of follow-up is shown in Table 3.

**Figure 2:**
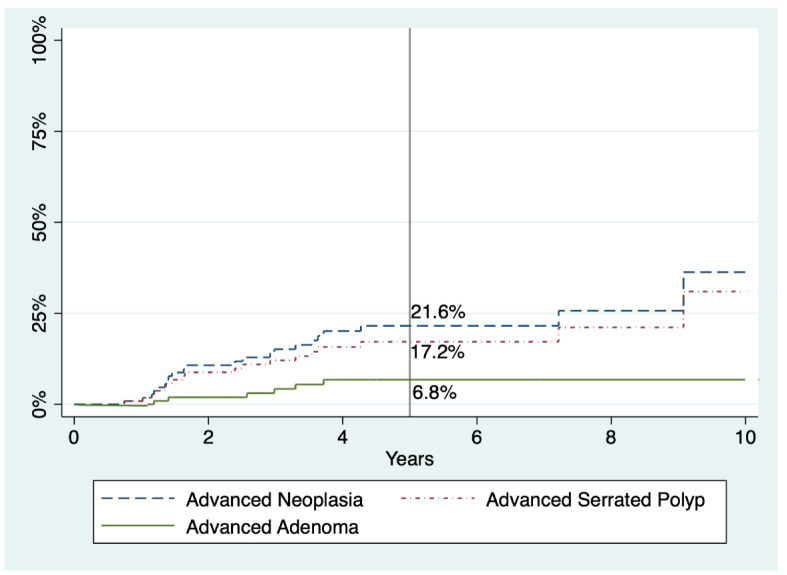
Cumulative incidences of advanced adenoma, advanced serrated polyp, and advanced neoplasia within follow-up in patients with SPS. Percentages refer to cumulative incidence at five years.

**Figure 3:**
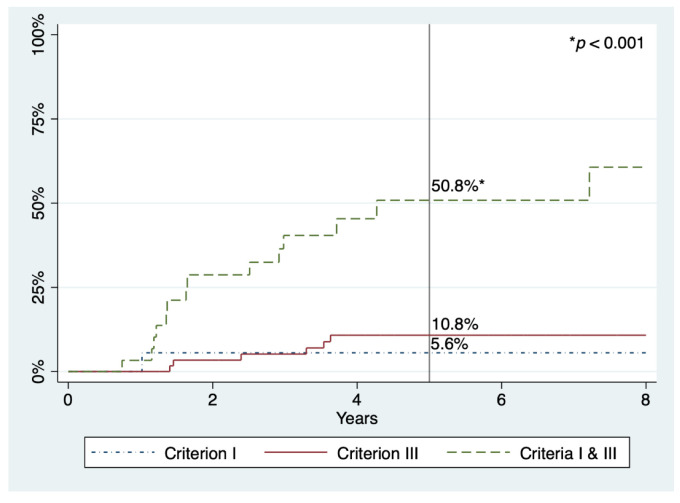
Cumulative incidences of advanced neoplasia within follow-up in patients with SPS, according to 2010 WHO criteria. Percentages refer to cumulative incidence at five years.

Regarding advanced SPs and AN, there seems to be a slight trend to decrease with time, but no statistical significance was reached in either case. The five-year cumulative incidences for advanced SP, advanced adenoma, and AN were 17.2%, 6.8%, and 21.6%, respectively (Table 4 and Figure 2). Finally, the five-year cumulative incidence specifically for AN according to WHO criteria was 5.6%, 10.8%, or 50.8% in patients who fulfilled criterion I, III, and both, respectively (*p* < 0.001) (Figure 3).

From the total of 109 patients, only 1 (0.9%) required surgery (ileo-caecal resection) at his 4th year of surveillance, due to an unresectable adenoma harbouring an intramucosal carcinoma (pTis). The subject fulfilled both 2010 WHO criteria I and III, having had advanced SPs before starting surveillance with the previous colonoscopy performed 13.6 months before, being complete and well prepared. The other 108 (99.1%) patients were managed endoscopically.

Seven patients were excluded due to any interval between colonoscopies of over 42 months. They all fulfilled WHO criterion III and there were neither prior occurrences of CRC nor colonic surgery. In 6 out of the 7, the delay was between the clearance and the first surveillance colonoscopy (median 4.5 years, IQR = 4.1–5.4) and in the other one between the 2nd and the 3rd. Eighteen colonoscopies were performed, with a median of two per patient (IQR = 1–4) and a median follow-up of 6.9 years (IQR = 5.8–9.6). Two advanced adenomas were resected at 5.3 and 6.8 years of follow-up, whereas neither CRC nor advanced SPs were found. We could manage all of these seven patients endoscopically.

## 3. Discussion

In this report, we present the results of surveillance of a large cohort of patients with SPS in a single centre. As far as we know, this is the study with the longest follow-up after colonic clearance, with some patients having been under surveillance since 2008. During a median of 5.0 years, we observed that 21.6% of patients developed AN at five years, whereas in our multicentre retrospective study—with a median follow-up of 2.2 years—this risk was over 50% [23]. The only prospective research that described explicitly the cumulative incidence of AN, including 271 patients followed for a median of 3.6 years, found it to be 44.0% at five years [25]. We also noticed that this risk was significantly higher in patients who fulfilled both 2010 WHO criteria I and III compared to those who met criterion I or III alone (50.8%, 5.6%, and 10.8%, respectively), which is consistent with previous studies [23,25]. However, there were few patients who only met criterion I in our cohort, thus their actual risk could have been underestimated.

The median interval between follow-up colonoscopies was 1.8 years as a result of the introduction in our centre of the personalized surveillance protocol proposed by Bleijenberg et al., compared to the 1.1 years in our previous work. Even so, the incidence of AN each year ranges from 0 to 8.3%. Looking closely at the per-patient incidence of advanced SPs and AN each year of surveillance (Table 3), we observed that, although not statistically significant, there seems to be a small tendency of a decrease over time. In fact, acknowledging the relatively few remaining patients at that point, we only diagnosed one AN within the 8th and the 15th year of follow-up. This trend was also described in the aforementioned personalized follow-up protocol. Furthermore, that study showed that individuals with prior surveillance colonoscopies had a lower risk of developing AN during the study than those who started endoscopic follow-up within the protocol (HR 0.64, 95%CI 0.41–0.99; *p* = 0.047) [25]. Conversely, other works reported a relatively constant incidence of AN during endoscopic surveillance, and we tried to explain the possible reasons elsewhere [23,24,27]. We speculate whether the absence of statistical significance in our curves might be due to the low incidence of advanced lesions since the beginning of surveillance, in contrast to prior studies, or to the lack of statistical power because of an insufficient number of participants.

We diagnosed CRC in a total of 11 patients in our initial group of 129, with only two of these occurring after 2014. This represents an 8.5% of our whole cohort, whereas other large studies reported figures ranging from 15.8% to 36.7% [21,22,25,27]. In any case, all reports demonstrated that the vast majority of CRC was diagnosed before or at the moment of SPS diagnosis. Interestingly, we did not find any invasive CRC during surveillance, and the most advanced lesion we diagnosed was an intramucosal carcinoma (pTis), resected surgically. The absence of CRC within endoscopic follow-up was also described in two retrospective studies, in a single-centre with 60 individuals under maintenance for a mean of 2.1 years [28] and a multicentre in which 96 patients were under surveillance for a median of 3.6 years [29]. Another research, a single-centre prospective protocol with annual colonoscopy found no incident CRC in 41 patients within a median of 3.1 years [24]. Some other works have found low five-year cumulative incidences of CRC, ranging from 1.0% to 1.9%, two retrospective [21,22] and two recent prospective focused only on surveillance, with 271 and 142 participants and median follow-ups of 3.6 and 3.9 years, respectively [25,27]. Finally, two retrospective multicentre reports with 77 and 152 individuals described cumulative incidences of 7.0% and 6.4% at five years, respectively [6,23], being considerably higher than the rest of the studies published. At least in our group’s previous work, we believe that a possible reason for these discrepancies could be the study’s retrospective design with 18 participating hospitals, with some of them recruiting few patients. This could have led to the latter centres to select individuals with a more aggressive SPS phenotype due to ascertainment bias.

We consider that several factors could explain the differences with previous reports. First, this is a single-centre study carried out in the setting of a high-risk clinic for digestive cancer with a special interest in SPS. This would lead to a quite homogeneous surveillance, following the current recommendations at all times. Second, we maintain a high level of clinical suspicion regarding this condition, so usually we identify patients at the very moment they fulfil any of the WHO criteria. This approach allowed us a thorough recruitment of patients, some of them immediately after diagnosis, thus minimizing ascertainment bias because even those with a less aggressive phenotype were included. In fact, 56.0% of our patients fulfilled only WHO criterion III compared to 35.2% and 36.5% in two different follow-up studies that reported global CRC risks of 36.7% and 25.5%, respectively [25,27]. Moreover, Carballal et al. found a 15.8% risk of CRC in a cohort with 45.3% of individuals who fulfilled criterion III alone [21]. In contrast, Parry et al. showed neither prevalent nor incident CRC in 96 patients, with 70.0% of them meeting only criterion III [29]. It has been already established that individuals who meet criterion III alone have less risk of CRC and AN [22,23], and we believe that the weight of this group in our cohort plays a relevant role in the low risk of both CRC and incident AN we found. Third, as a result of our early detection of patients with SPS, in most of them we started proper endoscopic follow-up soon in the natural history of the disease and this could have avoided the development of CRC in many cases, as suggested by Bleijenberg et al. [27]. Actually, 9 out of 11 CRC were detected before 2014, when SPS was frequently underdiagnosed. Fourth, we used high-definition technology in almost 90% of colonoscopies and chromoendoscopy—narrow band imaging (NBI) and/or dye-based—in more than two thirds. Even though there is no evidence that NBI improves polyp detection in SPS [30], we frequently used it for characterization and the better delineation of lesions. On the other hand, we performed 96 panchromoendoscopies (28.1%) with indigo carmine, which has been shown to increase the diagnostic yield of colonoscopy in this condition, mostly due to small SPs [31]. We also used Endocuff Vision^TM^ in a few colonoscopies, in the context of a clinical trial that did not prove its usefulness in SPS in any case [32]. The use of panchromoendoscopy with indigo carmine—mainly since 2015—could be one of the reasons why we found and resected a total of 2097 polyps in 342 surveillance colonoscopies, compared to the 1308 polypectomies in 447 colonoscopies from another long-term follow-up study [27]. Furthermore, the resection of small SPs, favoured by panchromoendoscopy with indigo carmine, would prevent the development of AN in subsequent colonoscopies, as we have seen.

As regards management of patients during follow-up, we only had to refer one of them (0.9%) for surgery because of an unresectable adenoma with an intramucosal carcinoma (Tis). This fact is consistent with previous studies, showing that the vast majority of patients with SPS can be managed endoscopically after colonic clearance [23,25,27,28,29].

Besides our main analysis, we would like to comment on the findings of the seven patients excluded for not having any colonoscopy for 42 months. In fact, in some patients this interval was beyond five years. Remarkably, during a median follow-up of 6.9 years, we did not find any CRC and only two advanced adenomas were resected.

Despite the new recommendations from the ESGE 2019 guidelines [26], the endoscopic surveillance of SPS still represents a considerable burden, not only for endoscopy units but also for patients. It has been recently proven that the extension of surveillance intervals in low-risk patients from one to two years is safe, and the authors discussed the possibility of further lengthening such intervals in those patients, to three or five years [25]. However, in a new long-term research involving only patients from their own centre, they observed a quite stable incidence of AN throughout endoscopic follow-up [27]. The authors speculated whether that incidence could be due to small polyps missed in prior colonoscopies or because of newly developed lesions. In any case, Bleijenberg et al. considered that their findings discouraged deintensifying surveillance even after several endoscopies. There is evidence nowadays that patients with SPS need long-term endoscopic follow-up due to their CRC and AN risk, and stratification according to their polyp burden is at the same time safe and effective to reduce the number of colonoscopies [25]. A question remains regarding whether it is still possible to extend safely the intervals between surveillance colonoscopies. We consider that our results support the safety for a further extension, at least in the low-risk group, with new intervals of three years or even longer. All in all, perhaps the main aim of surveillance should be to prevent the development of CRC, and to avoid surgery as far as possible, instead of focusing basically on AN. In such settings, we wonder if a relatively high (over 40%) incidence of AN—maintaining low rates of CRC—might be acceptable as a consequence of a significant reduction in colonoscopy burden. A more conservative option would be to lengthen the times between colonoscopies only after a certain number of them, considering that the incidence of AN might decrease in subsequent rounds. Regarding which patients may be candidates for these newly extended intervals, only the fulfilment of the 2010 WHO criterion III alone has been confirmed in different studies as a predictor of low risk for incident AN [23,25], but criteria proposed by Bleijenberg et al. have also proven useful.

We believe our work has some strengths. First, this is the series with the longest follow-up after clearance reported so far, with seven patients surveilled for 10 years or more. Second, our cohort is large enough to obtain reliable conclusions. Finally, we recruited participants thoroughly in the setting of a high-risk clinic for digestive cancer, including all different phenotypes of the disease. Nevertheless, we have to acknowledge several potential limitations as well. First of all, its retrospective enrolment, even though data collection and follow-up of patients were prospective. Second, as a single-centre study our conclusions may be more difficult to apply to the general population. On the other hand, this allowed for a more homogeneous surveillance of patients. Third, until 2015, when we were invited to participate in the study of Bleijenberg et al. [25], we did not follow a predetermined surveillance protocol, but adapted endoscopic follow-up to the current recommendations at all times which have changed during these 12 years. Fourth, although they are registered in our unit databases, we did not analyse complications related to surveillance colonoscopies, as other studies have done. Finally, our participants were selected according to 2010 WHO criteria instead of using those updated in 2019. This makes sense because follow-up started years ago, and in any case it is unlikely to have had any effect on the study considering we excluded patients who only met the extinct 2010 criterion II, and the other criteria are quite similar in both versions.

## 4. Materials and Methods

### 4.1. Study Population

Until October 2020, we retrospectively recruited from a prospectively collected database patients who fulfilled 2010 World Health Organization (WHO) criteria [7] for SPS. We ascertained SPS diagnosis using endoscopic and histopathological reports from all lesions resected at colonoscopy and/or surgery. All individuals aged 18 years or older with SPS followed at our high-risk clinic for digestive cancer in whom colonic clearance was performed were candidates to enter the study. We defined colonic clearance as the removal of all polyps >3 mm in a well-prepared colonoscopy with confirmed caecal intubation; sometimes more than one colonoscopy—performed within 6 months—and/or colonic surgery were needed to achieve clearance.

We excluded from the study patients with other high-risk conditions for CRC, such as inflammatory bowel disease, Lynch or Lynch-like syndromes, and known mutations in APC or MUTYH (biallelic); those who only fulfilled 2010 criterion II, abandoned in the 2019 updated criteria [8]; and individuals with a total colectomy at SPS diagnosis.

The Clinical Research Ethics Committee of the Hospital Universitario de Móstoles approved the study protocol, and all patients gave written informed consent.

### 4.2. Clinical and Demographic Characteristics

We collected data such as age, sex, height, weight, smoking habits, and personal history of CRC or other malignancies. We also registered information concerning SPS and/or tumours in first-degree and second-degree relatives. We obtained specific data regarding colorectal invasive tumours (size, location, morphology, differentiation grade, stage, etc.) from both endoscopic and histopathological reports.

### 4.3. Clinical, Endoscopic, Surgical, and Histopathological Records

Once SPS diagnosis was established, patients started follow-up in our high-risk clinic for digestive cancer, where endoscopic surveillance intervals were scheduled. Between 2008 and 2015, although in most cases surveillance intervals were of one year, some of them could extend up to three years because of the absence of high-quality evidence. However, in 2015 we were invited to join the protocol of personalized endoscopic surveillance of Bleijenberg et al. [25]. Therefore, since that moment we have scheduled colonoscopies with an interval of either one or two years according to the findings at the previous examination.

We included in the study individuals who underwent surveillance at an interval of 1–3 years (±6 months) after colonic clearance. Consequently, patients with a follow-up interval between two consecutive colonoscopies longer than 42 months were excluded from the main analysis. Colonoscopies were performed with high-definition technology in 89.8% of cases and chromoendoscopy was routinely used: narrow band imaging was used in 139 (40.6%) colonoscopies, panchromoendoscopy with indigo carmine in 82 (24.0%), and both in 14 (4.1%).

We registered indications for sending patients to surgery (presence of CRC, unresectable polyp or severe polyposis) and type of surgery (total colectomy/proctocolectomy or segmental resection). Polyp features included number, size, location, and pathology characteristics.

Tissue samples were routinely assessed by gastrointestinal pathologists. Serrated polyps were classified as hyperplastic polyps, sessile serrated lesions (previously known as “sessile serrated adenoma/polyps” or “sessile serrated polyps”), and traditional serrated adenoma, based on the WHO classification criteria [7]. Pathologists analysed SPs regarding the presence or absence of cytological dysplasia and, if present, it was either low or high-grade. Invasive cancer was defined as neoplastic extension vertically into the submucosal layer or beyond.

### 4.4. Outcome Measures

Regarding the location of polyps, we divided the colon in three segments: “proximal colon”, between the cecum and transverse colon; “descending colon”, splenic flexure and descending colon; and “distal colon”, sigmoid and rectum.

According to the literature, we defined: (a) advanced adenomas as those ≥10 mm in size, with villous components and/or high-grade dysplasia; (b) advanced SPs as those ≥10 mm in size and/or with dysplasia; and (c) AN as advanced SP, or advanced adenoma, or invasive CRC.

### 4.5. Statistical Analysis

Qualitative data are expressed as total numbers and frequencies (%), whereas quantitative data are expressed as means and standard deviations (SD), or medians and interquartile ranges (IQR). To analyse possible associations, we used a Chi-squared test in the case of qualitative variables and Student’s t test for quantitative variables. We obtained cumulative incidences of colorectal lesions by Kaplan–Meier survival analysis, calculating 95% confidence intervals (CIs). We compared survival curves using a Mantel–Haenszel (LogRank) test. We considered statistically significant *p* values < 0.05. We performed all analyses and graphs using Stata 16.1 (StataCorp LLC, College Station, TX, USA).

## 5. Conclusions

The risk of invasive CRC in patients with SPS during proper endoscopic follow-up seems to be very low, even in the long-term. Cumulative incidences of advanced colorectal neoplasia could be lower than previously described, at least in patients who only fulfil the 2010 WHO criterion III, even though a significant decrease of its incidence throughout the years has not been demonstrated. We have shown that the vast majority of patients can be managed endoscopically once colonic clearance is achieved. Our findings suggest that at least for the group of patients at lower risk there may be a benefit in the extension of intervals between surveillance colonoscopies. These benefits would be the reduction of both the risk of complications such as perforation or haemorrhage, and discomfort associated to colonic cleansing and to colonoscopy itself. Consequently, a new prospective research focused on the safety of longer follow-up intervals according to personal risk factors and polyp burden seems to be necessary.

## Figures and Tables

**Table 1:** Baseline characteristics of patients with SPS included in the surveillance (*n* = 109).

Demographic and Clinical Features	Values
Female, number (%)	53 (48.6)
Age at SPS diagnosis: years, mean (SD)	59.4 (8.9)
First degree relative with CRC, number (%)	42 * (38.5)
WHO SPS 2010 classification, number (%)	
I	18 (16.5)
III	61 (56.0)
I + III	30 (27.5)
Previous partial colonic surgery, number (%)	9 (8.3)
Appendicectomy	1 (11.1)
Right hemicolectomy	4 (44.4)
Left hemicolectomy	1 (11.1)
Sigmoidectomy	1 (11.1)
Lower anterior resection	2 (22.2)
Previous CRC, number (%)	9 (8.3)
Age at diagnosis: years, mean (SD)	59.4 (6.2)
Proximal to splenic flexure, number	4
Descending colon, number	0
Rectosigmoid, number	5

* 7 of them <50 years old.

**Table 2:** Surveillance colonoscopies (*n* = 109).

Colonoscopies and Polyps	Values
Total number of surveillance colonoscopies	342
Surveillance colonoscopies per patient: median (IQR)	3 (2–4)
Follow-up after clearing colonoscopy: years, median (IQR)	5.0 (3.3–7.2)
Interval between surveillance colonoscopies ^1^: years, median (IQR)	1.8 (1.6–2.1)
Polyps resected during surveillance: total number, median per patient (IQR)	2097, 14.0 (7.0–27.5)
Hyperplastic polyps: total number, median per patient (IQR)	1718, 11.0 (4.5–24.0)
Sessile serrated lesions: total number, median per patient (IQR)	57, 0 (0–0)
Traditional serrated adenomas: total number, median per patient (IQR)	0, 0 (0–0)
Adenomas ^2^: total number, median per patient (IQR)	322, 2.0 (0–4.0)

^1^ Refers to 92 patients with two or more surveillance colonoscopies. ^2^ 8 advanced.

**Table 3:** Findings in surveillance colonoscopies (per-patient analysis).

Years of Surveillance															
Patients with Any, *n* (%)	1st *n* = 109	2nd *n* = 98	3rd *n* = 91	4th *n* = 79	5th *n* = 60	6th *n* = 47	7th *n* = 36	8th *n* = 24	9th *n* = 16	10th *n* = 7	11th *n* = 3	12th *n* = 2	13th *n* = 2	14th *n* = 1	15th *n* = 1
CRC	0	0	0	0	0	0	0	0	0	0	0	0	0	0	0
Intramucosal CRC on adenoma	0	0	0	1 (1.3)	0	0	0	0	0	0	0	0	0	0	0
SSL	9 (8.3)	4 (4.1)	1 (1.1)	1 (1.3)	5 (8.3)	6 (12.8)	1 (2.8)	0	2 (12.5)	0	0	0	0	0	0
TSA	0	0	0	0	0	0	0	0	0	0	0	0	0	0	0
SP ≥ 10 mm	7 (6.4)	4 (4.1)	3 (3.3)	3 (3.8)	3 (5.0)	1 (2.1)	0	0	0	0	0	0	0	0	0
Distal * SP	51 (46.8)	64 (65.3)	38 (41.8)	31 (39.2)	33 (55.0)	21 (44.7)	15 (41.7)	9 (37.5)	9 (56.3)	3 (42.9)	2 (66.7)	1 (50)	2 (100)	0	1 (100)
Proximal SP	39 (35.8)	35 (35.7)	20 (22.0)	17 (21.5)	19 (31.7)	14 (29.8)	4 (11.1)	4 (16.7)	8 (50.0)	1 (14.3)	0	1 (50)	1 (50)	0	0
Proximal SSL	9 (8.3)	2 (2.0)	0	1 (1.3)	4 (6.7)	6 (12.8)	0	0	2 (12.5)	0	0	0	0	0	0
SP with dysplasia	0	0	0	0	0	1 (2.1)	1 (2.8)	0	1 (6.3)	0	0	0	0	0	0
Advanced SP	7 (6.4)	4 (4.1)	3 (3.3)	3 (3.8)	3 (5.0)	2 (4.3)	1 (2.8)	0	1 (6.3)	0	0	0	0	0	0
Advanced adenoma	2 (1.8)	0	4 (4.4)	1 (1.3)	0	0	0	0	0	0	0	0	0	0	0
Advanced neoplasia	9 (8.3)	4 (4.1)	6 (6.6)	4 (5.1)	3 (5.0)	2 (4.3)	1 (2.8)	0	1 (6.3)	0	0	0	0	0	0
Need for surgery **	0	0	0	1 (1.3)	0	0	0	0	0	0	0	0	0	0	0

* Distal to splenic flexure; ** Due to polyps.

**Table 4:** Cumulative incidence of lesions during follow-up.

Lesion	Three Years	Five Years
CRC	0%	0%
Intramucosal CRC on adenoma	0%	1.3% (95%CI 0–3.9)
SSL	12.9% (95%CI 6.3–19.5)	16.1% (95%CI 8.4–23.8)
TSA	0%	0%
SP ≥ 10 mm	12.1% (95%CI 5.6–18.5)	17.2% (95%CI 9.4–25.0)
Proximal SP	66.5% (95%CI 57.2–75.8)	78.5% (95%CI 70.0–87.0)
Proximal SSL	10.7% (95%CI 4.7–16.8)	13.9% (95%CI 6.6–21.1)
SP with dysplasia	0%	0%
Advanced SP	12.1% (95%CI 5.6–18.5)	17.2% (95%CI 9.4–25.0)
Advanced adenoma	4.2% (95%CI 0.2–8.3)	6.8% (95%CI 1.5–12.1)
Advanced neoplasia:		
Global	15.2% (95%CI 8.1–22.3)	21.6% (95%CI 13.1–30.1)
Criterion I (*n* = 18)	5.6% (95%CI 0–16.1)	5.6% (95%CI 0–16.1)
Criterion III (*n* = 61)	5.2% (95%CI 0–10.9)	10.8% (95%CI 2.7–19.0)
Criteria I+III (*n* = 30)	40.4% (95%CI 21.8–59.0)	50.8% (95%CI 30.6–71.1)

## Data Availability

The data are not publicly available due to our institutional policy.
